# Supplementary material for: A gateway conspiracy? Belief in COVID-19 conspiracy theories prospectively predicts greater conspiracist ideation
Source: PLoS One. 2022 Oct 26;17(10):e0275502. doi: 10.1371/journal.pone.0275502 (PMC9604008; doi:10.1371/journal.pone.0275502)
Supplement: S1 File — (DOCX) [file pone.0275502.s001.docx]

**Supplemental Material**

In this supplement, we report three sets of analyses. First, we report the results of procedures aimed at detecting the possible impact of attrition on the results reported in each of the two studies. Second, we examine the extent to which the analyses involving changes in conspiracist ideation reported in the main text remained substantively unchanged when individuating the two components of our measure of belief in COVID-19 conspiracy theories. Third, we test the degree to which the changes in generic conspiracist beliefs reported using aggregated scores on the Generic Conspiracist Beliefs Scale (Study 1; Brotherton et al., 2013) and the Conspiracy Mentality Questionnaire (Study 2; Bruder et al., 2013) were evident when individuating the items of the scale.

**Probing the Impact of Attrition**

**Study 1**

Despite our best efforts, the rate of attrition in Study 1 was substantial. This attrition is likely at least partly a result of the method of participant recruitment. Most relevant to the current discussion is the relatively high rate of turnover in MTurk’s participant population. It has been estimated that half of the participant pool leaves MTurk and is replaced approximately every seven months (Stewart et al., 2015). Thus, we believe that our attrition rate was influenced by the combination of constant turnover and the amount of time that elapsed between our waves of data collection (six months).

To assess the of the possible role of attrition in our study, we conducted a series of analyses. Following the recommendations of Miller and Hollist (2007), we determined that neither conspiracist ideation, *β =* -0.25, *SE* = 0.16, Wald = 2.51, *p* = 0.11, odds ratio = 0.78, nor belief in COVID-19 conspiracy theories, *β =* -0.28, *SE* = 0.17, Wald = 2.88, *p* = 0.09, odds ratio = 0.76, were significantly associated with attrition. Moreover, out of a large set of demographic and attitudinal variables included in the study, only age, *β =* 0.26, *SE* = 0.11, Wald = 5.69, *p* = 0.017, odds ratio = 1.29, and the perception that the threat of COVID-19 is exaggerated, *β =* -0.64, *SE* = 0.12, Wald = 26.0, *p* < 0.001, odds ratio = 0.53, significantly predicted attrition. Despite these mean-level differences between samples, there remained a considerable amount of variance in the follow-up sample in terms of both age (*SD =* 14.0) and perception of threat posed by COVID-19 (*SD* = 0.98 on a 1 to 4 scale). This considerable variability allowed us to test whether the relations reported in the main text were moderated by either age or perceptions of threat of COVID-19. The results revealed that age did not significantly moderate the relation between belief in COVID-19 conspiracy theories and belief in voter fraud in the 2020 American Presidential election, *β* = 0.15 (95% CI: -0.004, 0.28), *t*(101) = 1.93, *p* = 0.06. Despite this interaction not reaching statistical significance, we decided to probe it to understand the general nature of the relation. Simple slopes analyses revealed that among those who were older (1 *SD* above the mean), belief in COVID-19 conspiracy theories strongly predicted belief that there was voter fraud in the 2020 American presidential election, *β* = 0.58 (95% CI: 0.36, 0.79), *t*(105) = 5.37, *p <* 0.001. Among those who were relatively younger (1 *SD* below the mean), belief in COVID-19 conspiracy theories was a somewhat weaker predictor of belief in the voter fraud conspiracy theory, *β* = 0.30 (95% CI: 0.11, 0.49), *t*(105) = 3.06, *p* = 0.003. Thus, though the relation between belief in COVID-19 conspiracy theories and belief in the voter fraud conspiracy was somewhat smaller among younger people, it remained positive and statistically significant across age levels.

Moreover, age did not significantly moderate the relation between belief in COVID-19 conspiracy theories and change in generic conspiracist beliefs, *β* = -0.06 (95% CI: -.16, 0.043), *t*(101) = -1.16, *p* = 0.25. Similarly, perceptions of the threat posed by COVID-19 did not moderate the relation between belief in COVID-19 conspiracy theories and belief in voter fraud in the 2020 American election, *β* = -0.05 (95% CI: -0.18, 0.08), *t*(102) = -0.79, *p* = 0.43, nor did it moderate the relation between belief in COVID-19 conspiracy theories and change in generic conspiracist beliefs, *β* = -0.04 (95% CI: -.13, 0.05), *t*(102) = -0.82, *p* = 0.42.

Pursuing yet another recommendation offered by Miller and Hollist (2007), we next examined the role of attrition by comparing the matrix of baseline correlations among key variables for the overall sample (see Table 1) to that of those who participated in the follow-up wave of data collection (see Table 2). This comparison suggests that the subsample that participated in the follow-up wave of data collection was comparable to the full sample.

**Table 1.**

*Correlation matrix for those who participated in the baseline wave of data collection.*

| Variable | 1. | 2. | 3. | 4. | 5. |
| --- | --- | --- | --- | --- | --- |
| 1. Conspiracist ideation | - |  |  |  |  |
| 2. Belief in COVID-19 conspiracy theories | .71** | - |  |  |  |
| 3. Political orientation | .24** | .41** | - |  |  |
| 4. Belief that the threat of COVID-19 is exaggerated | -.46** | -.71** | -.37** | - |  |
| 5. Age | -.13** | -.08 | .09 | .09 | - |

*Note. N* = 441. ** indicates *p* < 0.01

**Table 2.**

*Correlation matrix for those who participated in the follow-up wave of data collection.*

| Variable | 1. | 2. | 3. | 4. | 5. |
| --- | --- | --- | --- | --- | --- |
| 1. Conspiracist ideation | - |  |  |  |  |
| 2. Belief in COVID-19 conspiracy theories | .68** | - |  |  |  |
| 3. Political orientation | .31** | .50** | - |  |  |
| 4. Belief that the threat of COVID-19 is exaggerated | -.34** | -.65** | -.37** | - |  |
| 5. Age | -.11 | -.06 | .04 | .08 | - |

*Note. N* = 107. ** indicates *p* < 0.01

**Study 2**

Participant retention across the three waves of data collection relevant to the current investigation was 51%—a rate that is higher than that of both Study 1 and comparable research projects (e.g., Czeisler et al., 2021). McBride and colleagues (2021) conducted a comprehensive investigation of the predictors of attrition in the project in question, and concluded that it was mostly attributable to demographic rather than psychological variables. To complement these findings, we conducted a series of additional analyses that focused on the variables most relevant to the current investigation. Most importantly, we found that attrition was not significantly related to either conspiracist ideation, *β =* -0.033, *SE* = 0.044, Wald = 0.54, *p* = 0.46, odds ratio = 0.97, or belief in COVID-19 conspiracy theories, *β =* 0.001, *SE* = 0.002, Wald = 0.001, *p* = 0.99, odds ratio = 1.0. Expanding beyond these central variables, we sought to determine whether attrition related to other factors associated with belief in conspiracy theories (e.g., political ideology) that may have introduced bias into our analyses. Out of a set of such associated psychological variables and demographic characteristics, only age significantly predicted attrition, *β =* 0.74, *SE* = 0.054, Wald = 185.00, *p* < 0.001, odds ratio = 2.09. Mirroring the procedure above intended to test whether the strength or direction of the gateway conspiracy effect differed as a function of age, we found no evidence for such an interaction, *β* = 0.035 (95% CI: -0.015, 0.086), *t*(1032) = 1.37, *p* = 0.17. Lastly, we compared the correlation matrix involving key variables for the whole sample (see Table 3) to that for the sample we analyzed (see Table 4). Once again, we found the subsample used for our analyses to be comparable to the full sample.

**Table 3.**

*Correlation matrix for those who participated in the first two waves of data collection.*

| Variable | 1. | 2. | 3. | 4. | 5. |
| --- | --- | --- | --- | --- | --- |
| 1. Conspiracist ideation | - |  |  |  |  |
| 2. Belief in COVID-19 conspiracy theories | .21** | - |  |  |  |
| 3. Age | -.05 | -.12** | - |  |  |

*Note. N* = 1406. ** indicates *p* < 0.01

**Table 4.**

*Correlation matrix for those who participated in all waves of data collection.*

| Variable | 1. | 2. | 3. | 4. | 5. |
| --- | --- | --- | --- | --- | --- |
| 1. Conspiracist ideation | - |  |  |  |  |
| 2. Belief in COVID-19 conspiracy theories | .22** | - |  |  |  |
| 3. Age | -.05 | -.12** | - |  |  |

*Note. N* = 1037. ** indicates *p* < 0.01

**Individuating Measure of Belief in COVID-19 Conspiracy Theories**

**Study 1**

We then examined whether the relations we report in the main text involving belief in COVID-19-specific conspiracy theories were driven solely by one of the two constituent items of the scale. To this end, we conducted the key analyses once again, but used each individual item of the scale as a focal predictor instead of the composite score.^^[[1]](#footnote-1)^^ To start, we focused on the belief that COVID-19 is intentionally misrepresented as dangerous. We regressed belief in voter fraud in the 2020 American Presidential election on generic conspiracist beliefs and political orientation in an initial step, and then entered belief that COVID-19 was intentionally misrepresented as dangerous in a subsequent step. As expected, belief in voter fraud was significantly predicted by political orientation, *β* = 0.62 (95% CI: 0.47, 0.76), *t*(104) = 8.10, *p* < 0.001, and generic conspiracist beliefs, *β* = 0.18 (95% CI: 0.006, 036.), *t*(104) = 2.44, *p* = 0.016. More importantly, greater belief that COVID-19 was intentionally misrepresented as dangerous predicted greater belief that there was voter fraud in the 2020 American Presidential election, *β* = 0.47 (95% CI: 0.30, 0.73), *t*(103) = 5.42, *p* < 0.001.

We then regressed generic conspiracist beliefs at follow-up on generic conspiracist beliefs at baseline and belief that COVID-19 was intentionally misrepresented as dangerous at baseline. Once again, greater generic conspiracist beliefs assessed at baseline significantly predicted greater generic conspiracist beliefs at follow-up, *β* = 0.68 (95% CI: 0.55, 0.84), *t*(104) = 11.16, *p* < 0.001. Above and beyond this effect, however, greater belief that COVID-19 was intentionally misrepresented as dangerous at baseline predicted greater generic conspiracist beliefs at follow-up, *β* = 0.26 (95% CI: 0.12, 0.38), *t*(104) = 4.24, *p* < 0.001.

Next, we turned our attention to the other COVID-19-specific conspiracy theory on which we focused—that COVID-19 was deliberately released into the world. Following the procedure detailed above, we found that greater belief that there was voter fraud in the 2020 American election was significantly predicted by higher levels of generic conspiracist beliefs, *β* = 0.18 (95% CI: 0.019, 0.35), *t*(104) = 2.48, *p* = 0.015, and greater conservatism, *β* = 0.61 (95% CI: 0.46, 0.76), *t*(104) = 8.21, *p* < 0.001, as well as greater belief that COVID-19 was deliberately released into the world, *β* = 0.36 (95% CI: 0.10, 0.62), *t*(103) = 3.78, *p* < 0.001. Moreover, greater belief that COVID-19 was deliberately released into the world prospectively predicted greater generic conspiracist beliefs, *β* = 0.41 (95% CI: 0.23, 0.54), *t*(104) = 6.89, *p* < 0.001, even when controlling for people’s level of generic conspiracist beliefs at baseline, *β* = 0.57 (95% CI: 0.45, 0.70), *t*(104) = 9.58, *p* < 0.001.

**Study 2**

For Study 2, we followed the same procedure as above for examining the impact of each item of our measure of belief in COVID-19 conspiracy theories on increases in generic conspiracist beliefs. Specifically, we regressed generic conspiracist beliefs at Wave 4 on generic conspiracist beliefs at Wave 1 in an initial step and belief that COVID-19 is no more dangerous than the flu in a subsequent step. Generic conspiracist beliefs at Wave 1 significantly predicted generic conspiracist beliefs at Wave 4, *β* = 0.60 (95% CI: 0.56, 0.65), *t*(1034) = 24.26, *p* < 0.001. As predicted, however, greater belief that the severity of COVID-19 has been exaggerated significantly predicted increases in generic conspiracist beliefs, *β* = 0.071 (95% CI: 0.014, 0.12), *t*(1034) = 2.85, *p* = 0.004.

Turning to the other item of the scale, we found that the belief that COVID-19 was created in a lab predicted increases in generic conspiracist beliefs over a period of several months, *β* = 0.18 (95% CI: 0.12, 0.24), *t*(1034) = 7.10, *p* < 0.001.

**Individuating the Measures of Generic Conspiracist Beliefs**

In the main text, we report that belief in COVID-19 conspiracy theories prospectively predicts increases in generic conspiracist beliefs. We wished to examine the degree to which the relation between belief in COVID-19 conspiracy theories and generic conspiracist beliefs was evident when evaluating the constituent items of our two measures of generic conspiracist beliefs singly. To do so, we first ran a series of regressions in which standing on each of the five items composing the trimmed version of the Generic Conspiracist Beliefs Scale administered at follow-up was predicted from standing on the corresponding item at baseline and the composite measure of belief in COVID-19 conspiracy theories at baseline (see Table 5). As expected, initial standing on generic conspiracist beliefs strongly predicted standing on the same dimension six months later across all items. Importantly, however, greater belief in COVID-19 conspiracy theories at baseline significantly predicted greater generic conspiracist beliefs at follow-up across all but one item—the belief that UFO sightings are staged to distract from real alien contact. Despite not reaching statistical significance, the direction of this relation was very much consistent with the significant relations observed for all other items.

We then repeated this same procedure using the data from Study 2 (see Table 6). Mirroring the findings above, initial standing on each item of the Conspiracy Mentality Questionnaire significantly predicted later standing, and belief in COVID-19 conspiracy theories predicted increases in all but one of the items of the questionnaire—though even the non-significant results was directionally-consistent with expectations.

**Table 5.**

*Predicting Change across Items of the Generic Conspiracist Beliefs Scale.*

| Outcome | Predictors | *β* | 95% CI | *t* | *p* |
| --- | --- | --- | --- | --- | --- |
| Government murders citizens – Follow-up | Government murders citizens – Baseline | .43 | (.26, .61) | 5.82 | < .001 |
|  | Belief in COVID-19 conspiracy theories | .44 | (.28, .58) | 6.03 | < .001 |
| Scientists deceive public – Follow-up | Scientists deceive public – Baseline | .25 | (.06, .43) | 9.37 | < .001 |
|  | Belief in COVID-19 conspiracy theories | .65 | (.45, .84) | 3.56 | .001 |
| Powerful people control world events – Follow-up | Powerful people control world events – Baseline | .44 | (.20, .65) | 6.10 | < .001 |
|  | Belief in COVID-19 conspiracy theories | .47 | (.30, .64) | 6.54 | <.001 |
| Staged UFO sightings distract from alien contact – Follow-up | Staged UFO sightings distract from alien contact – Baseline | .59 | (.40, .78) | 6.90 | < .001 |
|  | Belief in COVID-19 conspiracy theories | .12 | (-.30, .31) | 1.46 | .15 |
| Experiments are conducted without the public’s knowledge – Follow-up | Experiments are conducted without the public’s knowledge – Baseline | .36 | (.21, .53) | 4.63 | < .001 |
|  | Belief in COVID-19 conspiracy theories | .46 | (.31, .60) | 5.88 | < .001 |

*Note.* All variables were standardized prior to being entered into the model.

**Table 6.**

*Predicting Change across Items of the Conspiracy Mentality Questionnaire.*

| Outcome | Predictors | *β* | 95% CI | *t* | *p* |
| --- | --- | --- | --- | --- | --- |
| Public not informed about events – Wave 4 | Public not informed about events – Wave 1 | .46 | (.40, .52) | 16.60 | < .001 |
|  | Belief in COVID-19 conspiracy theories | .086 | (.034, .14) | 3.13 | .002 |
| Politicians hide motives – Wave 4 | Politicians hide motives – Wave 1 | .46 | (.39, .52) | 16.46 | < .001 |
|  | Belief in COVID-19 conspiracy theories | .039 | (-.021, .10) | 1.40 | .16 |
| Government monitors citizens – Wave 4 | Government monitors citizens – Wave 1 | .50 | (.44, .55) | 19.0 | < .001 |
|  | Belief in COVID-19 conspiracy theories | .21 | (.16, .26) | 7.92 | < .001 |
| Secret activities connect events – Wave 4 | Secret activities connect events – Wave 1 | .51 | (.46, .56) | 19.54 | < .001 |
|  | Belief in COVID-19 conspiracy theories | .20 | (.15, .25) | 7.72 | < .001 |
| Secret organizations influence decisions – Wave 4 | Secret organizations influence decisions – Wave 1 | .50 | (.44, .56) | 19.40 | < .001 |
|  | Belief in COVID-19 conspiracy theories | .21 | (.16, .27) | 8.25 | < .001 |

*Note.* All variables were standardized prior to being entered into the model.

**Discussion**

The results reported in this supplement clarify several key questions regarding our research. First, in neither study was attrition related to any of the key variables on which the research focuses. Moreover, despite finding mean-level differences in age and perceptions of threat posed by COVID-19 across the initial and follow-up sample in Study 1 and finding parallel differences involving age in Study 2, none of these variables significantly moderated the relations reported in the main text. Even when the results showed a trend toward an interaction, the simple effects showed that this weak difference between simple effects was a function of effect size, not direction of the relation. Importantly, there was no evidence that the nature of the relations between key variables differed between the full sample and those who participated in the follow-up wave of data collection in either Study 1 or Study 2. This consistency in correlations across measurement occasions offers yet another indication that attrition does not pose a serious threat to the internal validity of this study.

Second, we found that each item of our measure of belief in COVID-19 conspiracy theories significantly predicted belief in the voter fraud conspiracy in Study 1 and change in generic conspiracist beliefs over a period of several months in Studies 1 and 2. Thus, the relations reported in the main text involving belief in COVID-19 conspiracy theories are not a product of the specific content of one of the COVID-19 conspiracy theories used in either study.

Lastly, we found that the relation between belief in COVID-19 conspiracy theories and generic conspiracist beliefs was evident across almost all items of our measures of generic conspiracist beliefs—a five-item version of the Generic Conspiracist Beliefs Scale (Brotherton et al., 2013) and the Conspiracy Mentality Questionnaire (Bruder et al., 2013). Even among the two items for which this relation did not reach statistical significance, the direction of the regression coefficient was very much consistent with our predictions and the results of our other individual-item analyses. The near uniformity of these findings across items suggests that believing COVID-19 conspiracy theories has effects on conspiracist ideation that spans beyond topic-congruent generic conspiracist beliefs.

**References**

Brotherton, R., French, C. C., & Pickering, A. D. (2013). Measuring belief in conspiracy theories: The generic conspiracist beliefs scale. *Frontiers in Psychology*, *4*, 279.

Bruder, M., Haffke, P., Neave, N., Nouripanah, N., & Imhoff, R. (2013). Measuring individual differences in generic beliefs in conspiracy theories across cultures: Conspiracy Mentality Questionnaire. *Frontiers in Psychology*, *4*.

Czeisler, M. É., Wiley, J. F., Czeisler, C. A., Rajaratnam, S. M., & Howard, M. E. (2021). Uncovering survivorship bias in longitudinal mental health surveys during the COVID-19 pandemic. *Epidemiology and Psychiatric Sciences*, *30*.

McBride, O., Butter, S., Murphy, J., Shevlin, M., Hartman, T. K., Bennett, K. M., ... & Bentall, R. P. (2021). Design, content, and fieldwork procedures of the COVID‐19 Psychological Research Consortium (C19PRC) Study–Wave 4. *International Journal of Methods in Psychiatric Research*, e1899.

Miller, R., & Hollist, C. (2007). Attrition bias. In N. Salkind (Ed.). *Encyclopedia of Measurement and Statistics* (pp. 57-60). Sage Publications Inc.

Stewart, N., Ungemach, C., Harris, A. J., Bartels, D. M., Newell, B. R., Paolacci, G., & Chandler, J. (2015). The average laboratory samples a population of 7,300 Amazon Mechanical Turk workers. *Judgment and Decision making*, *10*(5), 479-491.

1. All variables were standardized prior to being entered into the regression. [↑](#footnote-ref-1)
